# TIPE2 Induced the Proliferation, Survival, and Migration of Lung Cancer Cells Through Modulation of Akt/mTOR/NF-κB Signaling Cascade

**DOI:** 10.3390/biom9120836

**Published:** 2019-12-06

**Authors:** Devivasha Bordoloi, Kishore Banik, Ganesan Padmavathi, Rajesh Vikkurthi, Choudhary Harsha, Nand Kishor Roy, Anuj Kumar Singh, Javadi Monisha, Hong Wang, Alan Prem Kumar, Ajaikumar B Kunnumakkara

**Affiliations:** 1 Cancer Biology Laboratory and DAILAB, DBT-AIST International Center for Translational and Environmental Research (DAICENTER), Department of Biosciences and Bioengineering, Indian Institute of Technology Guwahati, Guwahati, Assam 781039, India; devivasha@iitg.ac.in (D.B.); kishore.banik@iitg.ac.in (K.B.); padmavathi@iitg.ac.in (G.P.); rajes174106009@iitg.ac.in (R.V.); harsha.choudhary@iitg.ac.in (C.H.); r.nand@iitg.ac.in (N.K.R.); anujsingh@iitg.ac.in (A.K.S.); j.monisha@iitg.ac.in (J.M.); 2 Department of Pharmacology, Yong Loo Lin School of Medicine, National University of Singapore, Singapore 117600, Singapore; snrwh@nus.edu.sg; 3 Singapore Nuclear Research and Safety Initiative, National University of Singapore, Singapore 138602, Singapore; 4 Cancer Science Institute of Singapore, National University of Singapore, Singapore 117599, Singapore

**Keywords:** lung cancer, TIPE2, biomarker, Akt/mTOR, NF-κB, tobacco

## Abstract

Lung cancer represents the most common cause of cancer deaths in the world, constituting around 11.6% of all new cancer cases and 18.4% of cancer-related deaths. The propensity for early spread, lack of suitable biomarkers for early diagnosis, as well as prognosis and ineffective existing therapies, contribute to the poor survival rate of lung cancer. Therefore, there is an urgent need to develop novel biomarkers for early diagnosis and prognosis which in turn can facilitate newer therapeutic avenues for the management of this aggressive neoplasm. TIPE2 (tumor necrosis factor-α-induced protein 8-like 2), a recently identified cytoplasmic protein, possesses enormous potential in this regard. Immunohistochemical analysis showed that TIPE2 was significantly upregulated in different stages and grades of lung cancer tissues compared to normal lung tissues, implying its involvement in the positive regulation of lung cancer. Further, knockout of TIPE2 resulted in significantly reduced proliferation, survival, and migration of human lung cancer cells through modulation of the Akt/mTOR/NF-κB signaling axis. In addition, knockout of TIPE2 also caused arrest in the S phase of the cell cycle of lung cancer cells. As tobacco is the most predominant risk factor for lung cancer, we therefore evaluated the effect of TIPE2 in tobacco-mediated lung carcinogenesis as well. Our results showed that TIPE2 was involved in nicotine-, nicotine-derived nitrosamine ketone (NNK)-, N-nitrosonornicotine (NNN)-, and benzo[a]pyrene (BaP)-mediated lung cancer through inhibited proliferation, survival, and migration via modulation of nuclear factor kappa B (NF-κB)- and NF-κB-regulated gene products, which are involved in the regulation of diverse processes in lung cancer cells. Taken together, TIPE2 possesses an important role in the development and progression of lung cancer, particularly in tobacco-promoted lung cancer, and hence, specific targeting of it holds an enormous prospect in newer therapeutic interventions in lung cancer. However, these findings need to be validated in the *in vivo* and clinical settings to fully establish the diagnostic and prognostic importance of TIPE2 against lung cancer.

## 1. Introduction

Lung cancer represents one of the most prevalent cancers in the world, which develops in a multi-stage process via a series of genetic and epigenetic variations in the lung epithelial cells [1,2,3,4,5,6,7]. It constitutes approximately 11.6% of all new cancer cases and around 18.4% of total cancer-related deaths across the globe [8]. Notably, it is the leading cause of mortality due to cancer in men and one of the most fatal cancers among women [9,10]. The risk of lung cancer is reported to be 1:13 in males and 1:16 in females [11]. Besides, around 30-fold variation is observed in lung cancer mortality rates of males and females as well, which can be attributed to the trend of smoking, as tobacco smoking contributes to 90% of the lung cancer cases globally [12,13,14]. In India, lung cancer comprises 6.9% of new cancer incidences and 9.3% of cancer-related mortality in both the genders [15]. Lung cancer is broadly classified into two major classes on the basis of histological, clinical, and neuroendocrine characteristics, such as non-small cell lung cancer (NSCLC) and small cell lung cancer (SCLC). NSCLC represents the most genomically diverse type, constituting around 80–85% of total lung cancer cases and is associated with challenges in prevention and treatment strategies [1,16,17,18].

TIPE2 (tumor necrosis factor-α-induced protein 8-like 2), a cytoplasmic protein comprising of 184 amino acids, is a recently identified protein involved in the negative regulation of innate and cellular immunity [19]. It maintains immune homeostasis and is reported to be highly expressed in inflamed nervous tissue [20]. In addition, TIPE2 was found to be expressed in various cells, such as neurons in the brain and brainstem, hepatocytes, squamous epithelial cells in the esophagus and cervix, glandular epithelial cells in the appendix, colon, and stomach, and transitional epithelial cells in the ureter and bladder [21]. The crystal structure of human TIPE2 revealed it to comprise of six antiparallel α-helices, of which, α5 helix possesses a kink due to the presence of Pro153. Besides, TIPE2 bears a centrally located cylindrical cavity, mostly hydrophobic in nature, which is speculated as a cofactor binding site, whereas the outer surface of TIPE2 is found to be highly charged [20].

Over the last several years, a few studies have been carried out to evaluate the potential of TIPE2 as a clinical biomarker against different cancer types. For instance, one such study showed that TIPE2 prevented the migration and invasion of breast cancer cells through inhibition of β-catenin, cyclin D1, c-Myc, and epithelial-to-mesenchymal transition (EMT) [22]. Further, overexpression of TIPE2 prevented hypoxia-induced migration as well as invasion, and suppressed the expression of β-catenin, c-Myc, cyclin D1, and EMT in glial cells [23]. In addition, TIPE2 also suppressed the metastasis of gastric cancer cells through the activation of Glycogen synthase kinase 3 beta (GSK3β) and the inhibition of Akt [24]. It also downregulated Snail1 and Snail2/Slug in a GSK-3β- and proteasome-dependent manner through Akt in gastric cancer cells [25]. Moreover, adenovirus-mediated human TIPE2 gene transfer (AdVTIPE2) led to the suppression of gastric cancer cells’ growth through decreased Akt, ERK1/2, and activation of the intrinsic apoptotic pathway [26]. Besides, TIPE2 suppressed TNF-α-mediated metastasis of hepatocellular carcinoma (HCC) cells by inhibiting nuclear factor kappa B (NF-κB) and Erk1/2, indicating TIPE2 as a plausible target against HCC metastasis [27]. Further, in the case of NSCLC tissues, TIPE2 was found to be upregulated, which exerted a negative correlation with primary tumor size, lymph node metastasis, and advanced clinical stage of the disease [28]. In addition, TIPE2 overexpression led to decreased migration, invasion, and EMT in prostate cancer cells through PI3K/Akt inhibition [29].

In the present study, we evaluated the expression of TIPE2 in lung cancer tissues and its role in different processes involved in the development and progression of lung cancer. We found that TIPE2 plays a pivotal role in different processes of lung cancer development, such as survival, proliferation, invasion, and migration. It is also found to be involved in tobacco-mediated lung cancer. In addition, the underlined molecular mechanism of action is also elucidated.

## 2. Materials and Methods

### 2.1. Tissue Microarray

Expression of TIPE2 in normal lung tissues and different stages of lung cancer tissues was determined with the help of immunohistochemical analysis using tissue microarray (TMA) containing paraffin-embedded normal and malignant lung tissues (US Biomax, Inc., Cat. No. LC1503, Derwood, MD, USA). The TMA slide contained a total of 75 tissues, 150 cores (duplicated cores from the same patient in all cases) from different individuals: 29 adenocarcinoma, 3 adenosquamous carcinoma, 29 squamous cell carcinoma, 2 bronchioalveolar carcinoma, 4 small cell undifferentiated carcinoma, 2 large cell carcinoma, 1 neuroendocrine carcinoma, and 5 normal lung tissues (Table S1).

### 2.2. Immunohistochemistry

A Histostain-Plus Immunohistochemistry (IHC) Kit, HRP, broad spectrum (Invitrogen, Cat. No. 859043; Pleasanton, CA, USA) and a Metal-Enhanced DAB Substrate Kit (Cat No. 34065; Invitrogen, CA, USA) were used for immunostaining the TMA. Immunohistochemistry (IHC) was performed as per the manufacturer’s protocol, which includes deparaffinization, rehydration, peroxidase quenching, blocking, incubation with primary antibody and peroxidase conjugate secondary antibody, addition of DAB chromogen, and counterstaining with hematoxylin. Subsequently, the slide was dehydrated and mounted with coverslip using a D.P.X. mountant (Cat No DC4DF64352; Merck, Branchburg, NJ, USA). Anti-TIPE2 primary antibody (Cat. No. ab110389) was obtained from abcam^®^, Cambridge, MA, USA and used in the dilution of 1:50 for immunohistochemical analysis. The immunostained microarray slide was analyzed under an Olympus light microscope. Tissues that are stained brown are considered as positive for the presence of antigen of interest and given a score as per the staining intensity and number of positive cells. The score for the percentage of positive cells is scaled from 0 to 4+ and staining intensity is scaled from 1 to 3 [30,31,32].

### 2.3. The Cancer Genome Atlas (TCGA) Dataset Analysis

Information regarding the genetic alteration of TIPE2 in NSCLC patient samples was obtained from the open data portal of The Cancer Genome Atlas (TCGA) and cbioportal platforms (http://www.cbioportal.org) [33,34]. Prognosis of different NSCLC patients associated with the alterations of TIPE2 was evaluated in terms of disease/progression free survival (DFS/PFS) by the Kaplan–Meier survival curve [33,34].

### 2.4. Cell Culture

NCIH460 human NSCLC cells were procured from National Centre for Cell Science (NCCS), Pune, India. The cells were maintained in Dulbecco’s Modified Eagle Medium (DMEM; Gibco™; Life Technologies, Brooklyn, NY, USA), supplemented with 10% fetal bovine serum (FBS; Gibco^®^, Brooklyn, NY, USA) and 1X Pen-Strep (Invitrogen, CA, USA). The cells were cultured and maintained in a CO_2_-regulated incubator (37 °C, 5% CO_2_, and 95% humidity).

### 2.5. CRISPR/Cas9-Mediated Gene Knockout

In order to disrupt the TIPE2 gene, we used the CRISPR/Cas9 (Clustered regularly interspaced short palindromic repeats/CRISPR associated protein 9)-mediated gene editing method. CRISPR/Cas9 All-in-One Lentivector sets (Human) expressing both human Cas9 and respective single guide RNAs (sgRNAs), such as scrambled sgRNA CRISPR/Cas9 All-in-One Lentivector (Cat. No. K010) and TNFAIP8L2 (TIPE2) sgRNA CRISPR/Cas9 All-in-One Lentivector set (Human) (Cat. No. K2414705) were obtained from Applied Biological Materials, Richmond, BC, Canada. The sequences of sgRNA targets are given in Table S2. Initially, NCIH460 cells were seeded at a density of 25,000 cells/well, allowed to attain confluency by 70–80%, and then transfected with 1 µg of respective plasmids with the help of Lentifectin™ transfection reagent (Cat. No. G074, Applied Biological Materials, Richmond, BC, Canada) in incomplete opti-MEM media. After 5–8 h, 10% FBS (Gibco^®^, NY, USA) was added to the transfected cells. After 24 h, the media containing plasmid was replaced with fresh DMEM medium (with 10% FBS and 1X Pen-Strep). Following 24 h recovery, the positive selection of cells was carried out by adding 2.5 µg/mL of puromycin (Cat. No. P8833, Sigma-Aldrich, St. Louis, MO, USA). Confirmation of knockout of the selected clones was done with the help of Western blot analysis. 

### 2.6. Cell Viability Assay

The effect of TIPE2 knockout on the viability of lung cancer cells was determined with the help of an MTT assay. Briefly, the scrambled sgRNA transfected cells (represented as CRISPR/Cas9 scramble) and TIPE2 knockout cells (represented as CRISPR/Cas9 TIPE2) were seeded at a density of 2 × 10^3^ cells/well in 96-well plates and incubated at 37 °C in a CO_2_ incubator for 24 h. The MTT assay was performed at 0 and 72 h. After each time point, 3-(4,5-dimethylthiazol-2-yl)-2,5-diphenyl tetrazolium bromide; MTT (Cat. No. M2128, Sigma-Aldrich, MO, USA) was added and incubated for 2 h. After that, the culture medium was removed and 100 µl of DMSO (Cat No. 1.16743.0521, Merck, Darmstadt, Germany) was added and incubated at room temperature (RT) for 1 h. Finally, absorbance was measured at 570 nm with a microplate reader (TECAN Infinite 200 PRO multimode reader, Männedorf, Switzerland). The effect on viability caused due to the knockout of TIPE2 was then calculated by normalizing the absorbance value of 72 h with 0 h while taking the absorbance of CRISPR/Cas9 scramble as 100%. Further, the effect on the viability of tobacco components’-treated TIPE2 knockout cells was also evaluated using this assay, in which, after 24 h incubation of the seeded cells, four different tobacco components such as nicotine (1 µM), nicotine-derived nitrosamine ketone; NNK (0.05 µM), N-nitrosonornicotine; NNN (0.05 µM), and benzo[a]pyrene; BaP (0.25 µg/mL) were added to the CRISPR/Cas9 scramble as well as CRISPR/Cas9 TIPE2 cells. Nicotine (Cat No. N3876), NNK (Cat No. 78013), NNN (Cat No. 75285), and BaP (Cat No. B1760) were purchased from Sigma-Aldrich, St. Louis, MO, USA. The MTT assay was done at 0 and 24 h after adding the tobacco components and the same procedure was followed as previously mentioned. Finally, the reduction in viability of tobacco components’-treated TIPE2 knockout cells was measured by normalizing the absorbance value of 24 h with 0 h while considering the absorbance of CRISPR/Cas9 scramble treated with the respective tobacco components as 100%.

### 2.7. Colony Formation Assay

The clonogenic potential of TIPE2 knockout NCIH460 cells was determined with the help of a colony formation assay. CRISPR/Cas9 scramble and CRISPR/Cas9 TIPE2 cells were seeded at a low density (~1000 cells/well) and were allowed to grow for 2 weeks with replenishing of media when required. The colonies formed were fixed with 70% ethanol and then stained with 0.01% (*w*/*v*) crystal violet (Cat No: 548-6209; SRL Pvt. Ltd., Mumbai, India). The images of each well were captured, the individual clone types were identified, and the survival fraction was calculated. Moreover, the clonogenic potential of tobacco components’-treated TIPE2 knockout cells was also determined using this assay, in which, after 24 h incubation of the seeded cells, different tobacco components such as nicotine (1 µM), NNK (0.05 µM), NNN (0.05 µM), and BaP (0.25 µg/mL) were added to the CRISPR/Cas9 scramble as well as CRISPR/Cas9 TIPE2 cells. After incubating for 24 h, the media of all the wells were changed, cells were allowed to grow, and the same procedure was followed thereafter.

### 2.8. Migration Assay

This assay was carried out to determine the migration potential of NCIH460 cells after knockout of TIPE2 compared to the scrambled control. Initially, CRISPR/Cas9 scramble and CRISPR/Cas9 TIPE2 cells were seeded at a density of 6 × 10^5^ cells/well. When the formation of monolayer occurred, the medium was replaced with serum-free DMEM medium and incubated for 6–8 h. Subsequently, a wound was scratched in the culture well and then the migration of the cells was evaluated by observing the difference in the area of the wounds using an inverted microscope (Nikon T1-SM, Tokyo, Japan). Images were taken at different time intervals and then analyzed using Image J software. This assay was also performed to evaluate the effect of tobacco components on the migration potential of TIPE2 knockout cells. In case of that, after serum starvation followed by scratching of the wound, different tobacco components such as nicotine (1 µM), NNK (0.05 µM), NNN (0.05 µM), and BaP (0.25 µg/mL) were added to the CRISPR/Cas9 scramble as well as CRISPR/Cas9 TIPE2 cells and the migration potential of the cells was determined.

### 2.9. Cell Cycle Analysis

Cell cycle analysis was done to determine the effect of TIPE2 knockout on cell cycle progression of lung cancer cells. Briefly, CRISPR/Cas 9 scramble and CRISPR/Cas9 TIPE2 cells were seeded at a density of 1 × 10^5^ cells/well. After 24 h, cells were trypsinized, fixed with 75% ethanol at −20 °C overnight, and then treated with Propidium Iodide/ Ribonuclease (PI/RNase) solution (Cat No. A35126, Invitrogen) followed by incubation for 20 min in the dark. Subsequently, 25,000 cells in each sample were analyzed using a flow cytometer (FACS Celesta, Becton-Dickinson, Franklin Lakes, NJ, USA). The data obtained were then analyzed using FCS express (De Novo Software, Glendale, CA, USA) and finally, the cell count in different phases of the cell cycle was determined.

### 2.10. Western Blot

Western blot analysis was carried out for the confirmation of TIPE2 knockout in NCIH460 cells. Further, it was also done to determine different targets of TIPE2. Briefly, CRISPR/Cas9 scramble and CRISPR/Cas9 TIPE2 cells were lysed using whole cell lysis buffer containing protease inhibitors. The protein concentrations of the lysates were measured with the help of Bradford reagent (Cat. No. 500-0205; Bio-Rad, Hercules, CA, USA). Then 50 μg of proteins were resolved after mixing with 5X Laemmli Buffer in a 12% or 8% sodium dodecyl sulfate (SDS)-acrylamide gel. Subsequently, they were transferred to nitrocellulose membrane (Bio-Rad, CA, USA) and blocked, followed by probing of the blots with primary antibodies overnight (Table S3). Following this, the blots were incubated with appropriate horseradish peroxidase (HRP)-conjugated secondary antibodies (Table S3). The bands representing different proteins were visualized with the help of Clarity Western ECL Substrate (Cat. No. 1705061; Bio-Rad, CA, USA) in a ChemiDoc™ XRS System (Bio-Rad, California, USA). The house-keeping gene, α-tubulin, served as the loading control. Further, to determine the expression of different targets in tobacco components’-treated TIPE2 knockout cells, Western blot analysis was performed, in which lysis of the cells was done after 24 h of treatment with the tobacco components, such as nicotine (1 µM), NNK (0.05 µM), NNN (0.05 µM), and BaP (0.25 µg/mL), and the same process was carried out henceforth. 

### 2.11. Statistical Analysis

Statistical analysis was performed using Student’s *t*-test. All the data are represented as mean ± standard error (SE). *p*-value < 0.05 was denoted as statistically significant.

## 3. Results

In the present study, we determined the role of TIPE2 in lung cancer. Initially, we determined the expression of TIPE2 in lung cancer tissues through immunohistochemical analysis of TMA slides containing tissues of different lung cancer pathologies, stages, and grades. In addition, we elucidated the role of TIPE2 on different regulatory processes in lung cancer and underlined molecular mechanism of action. Besides, the role of TIPE2 in tobacco-promoted lung cancer was also determined.

### 3.1. Tumor Necrosis Factor-α-Induced Protein 8-Like 2 (TIPE2) is Upregulated in Human Lung Cancer

**Figure 1:**
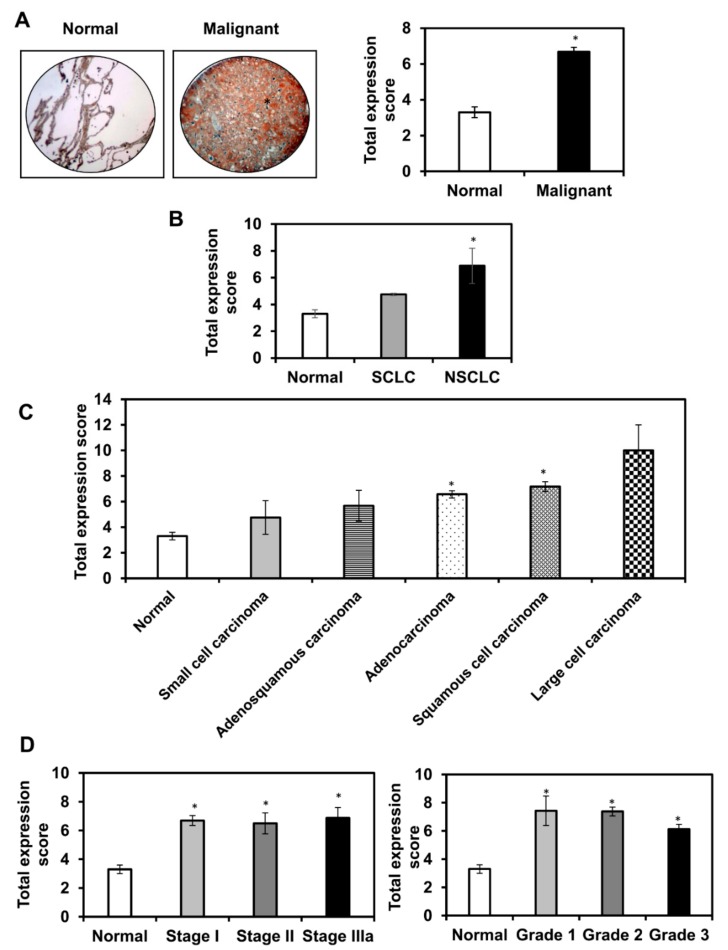
Expression of tumor necrosis factor-α-induced protein 8-like 2 (TIPE2) in normal and malignant lung tissues through immunohistochemical analysis. (**A**) Representative images of the expression of TIPE2 in lung cancer tissues (left panel). Expression of TIPE2 in normal versus malignant lung cancer tissues (right panel); (**B**) Expression of TIPE2 in two major lung cancer types: NSCLC: non-small cell lung cancer and SCLC: small cell lung cancer; (**C**) Expression of TIPE2 in different pathological conditions of lung cancer: small cell carcinoma, adenosquamous carcinoma, adenocarcinoma, squamous cell carcinoma, and large cell carcinoma; (**D**) Expression of TIPE2 in different stages of lung cancer (Left panel) and different grades of lung cancer (Right panel). Data are mean ± SE, * denotes *p* value < 0.05.

To understand the role of TIPE2 in lung cancer, we initially analyzed the expression of TIPE2 in lung cancer tissues. Our analysis revealed that TIPE2 was significantly upregulated in lung cancer tissues compared to normal lung tissues. Around 2-fold increase in the expression of TIPE2 was observed in the malignant lung tissues compared to the normal lung tissues. Thus, it provides an indication that TIPE2 might be involved in mediating malignant transformation of lung tissues (Figure 1A). Further, expression analysis of TIPE2 in normal, SCLC, and NSCLC tissue samples showed that TIPE2 was upregulated in both SCLC and NSCLC tissues compared to normal tissues, with more pronounced and significant upregulation in the NSCLC type (Figure 1B). In addition, upon comparing the differential expression of TIPE2 with respect to disease pathology, it was observed that TIPE2 exerted significant upregulation in adenocarcinoma and squamous cell carcinoma. Further, TIPE2 displayed around 2-fold increase in its expression in adenosquamous cell carcinoma tissues, whereas it exerted around 3-fold increase in its expression in large cell carcinoma tissues compared to the normal human lung tissues (Figure 1C). In addition, TIPE2 was found to be upregulated in different stages of lung cancer, such as stage I, II, and IIIa, and also in different grades of lung tumor, such as grade 1, 2, and 3, compared to normal lung tissues (Figure 1D).

### 3.2. Genetic Alteration of TIPE2 was Associated with Poor Diseas/Progression-Free Survival (DFS/PFS) of Non-Small Cell Lung Cancer (NSCLC) Patients

**Figure 2:**
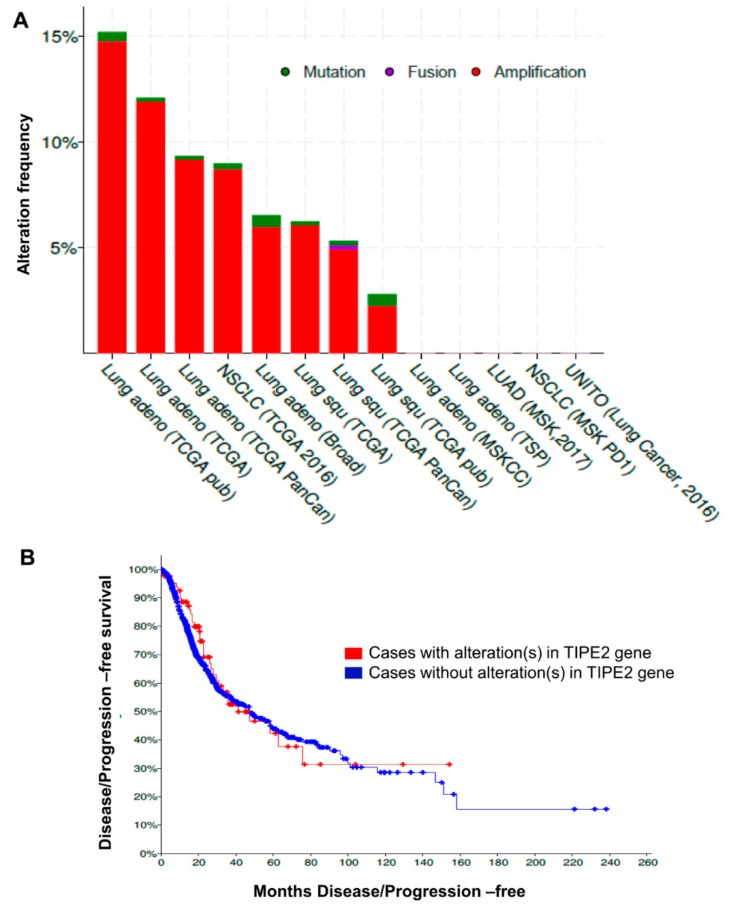
Mutational status of TIPE2 in NSCLC samples and its association with disease/progression- free survival (DFS/PFS) of NSCLC patients. (**A**) different types of genetic alterations of TIPE2 present in 1144 patients with NSCLC obtained from The Cancer Genome Atlas (TCGA) datasets. (**B**) Correlation of genetic alterations of TIPE2 and disease/progression free survival (DFS/PFS) of NSCLC patients.

The mutational status of TIPE2 in tissues of different NSCLC cancer patients was studied. Different types of genetic alterations, such as mutation, fusion, and amplification in 1144 patients with NSCLC were obtained and analyzed from TCGA datasets, and 16% genetic alteration was found to be present in TIPE2. While considering the univariate analysis for survival data of 1144 NSCLC patients from TCGA datasets, it was observed that the increasing copiousness of genetic alterations of the TIPE2 was associated with decreased DFS/PFS of NSCLC patients (Figure 2A,B). 

### 3.3. Knockout of TIPE2 Reduced the Viability and Survival of Lung Cancer Cells

**Figure 3:**
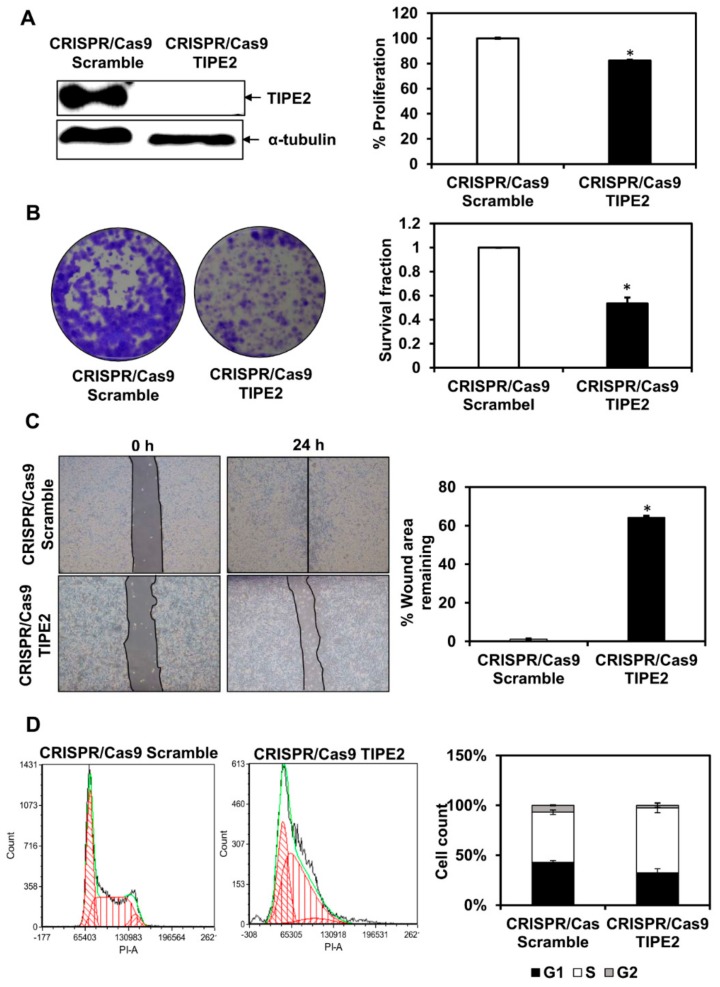
Knockout of TIPE2 in lung cancer cells and its effect on different cancer hall marks. (**A**) Western blot analysis showing the expression of TIPE2 in NCIH460 lung cancer cells after CRISPR/Cas9 knockout (Left panel); Effect of TIPE2 knockout on the viability of NCIH460 lung cancer cells as analyzed by MTT assay (Right Panel); (**B**) Colony formation assay showing decreased clonogenic potential of lung cancer cells after knockout of TIPE2 (Left panel). Graphical representation of decreased clonogenic potential of TIPE2 knockout cells in terms of survival fraction (Right panel). (**C**) Cell migration was detected with the help of wound healing assay. Images were taken at 10× magnification at 0 and 24 h (Left panel). Graphical representation of the decreased migration potential of TIPE2 knockout cells compared to CRISPR/Cas9 scramble (right panel). (**D**) Effect of TIPE2 knockout on the progression of cell cycle in lung cancer cells analyzed through flow cytometry using PI/RNase solution (Left panel). Graphical representation of the effect of TIPE2 knockout on the cell cycle progression of lung cancer cells (Right panel). Data are represented as mean ± SE, * denotes *p* value < 0.05.

Increased proliferation and survival are some of the key characteristics exhibited by cancer cells which are attained via modulation of different signaling cascades [35]. Therefore, to determine the effect of TIPE2 on the viability and survival of human lung cancer cells, first, knockout of TIPE2 was done. From the MTT assay, we observed that knockout of TIPE2 reduced the viability of NCIH460 cells compared to the scrambled control (Figure 3A). In addition, to determine the effect of TIPE2 knockout on the survival of NCIH460 cells, a colony formation assay was performed. This assay determines the clonogenic potential of cells, which can be described as the cell’s ability to proliferate indefinitely and retain its reproducibility to form a large colony which provides the measure of cell survival fraction [36]. The results showed that knockout of TIPE2 led to the reduced clonogenic potential of NCIH460 cells compared to the scrambled control, implying the involvement of TIPE2 in increasing the survival of lung cancer cells (Figure 3B). 

### 3.4. Knockout of TIPE2 Reduced the Migration of Lung Cancer Cells

In lung cancer, the most fatal cancer type in the world, the majority of patients are reported to have an extremely advanced disease stage. The ability of lung cancer cells to migrate and invade nearby cells is associated with their high metastatic potential [37,38]. Increasing lines of evidence suggest that diverse signaling molecules present in the tumor microenvironment play a vital role in the regulation of the migration of cancer cells [39]. Therefore, in order to know the involvement of TIPE2 in the modulation of the migration of lung cancer cells, the wound healing assay was performed. The results showed that loss of TIPE2 effectively reduced the migration potential of lung cancer cells. In case of the scrambled control, almost complete healing of the wound was observed at 24 h, whereas in TIPE2 knockout cells, more than 60% of the wound area remained at 24 h. Thus, TIPE2 is found to be involved in the modulation of lung cancer cells’ migration as well (Figure 3C).

### 3.5. Knockout of TIPE2 Led to the Arrest in the S phase of the Cell Cycle

Progression of the cell cycle is controlled through different regulatory points of various phases of the cell cycle, and deregulation of any of them can result in abnormal growth or apoptosis of cells [40]. Tumor cells generally display different molecular changes, which include overexpression of cyclins, cyclin-dependent kinases (CDKs), loss of CDK inhibitors, and tumor-suppressor proteins such as p53 due to epigenetic inactivation or gene mutations [41]. In the case of cell cycle analysis, we found that knockout of TIPE2 led to an increase in the number of cells in S phase compared to CRISPR/Cas9 scramble lung cancer cells. The arrest in the S phase of the cell cycle suggested that knockout of TIPE2 plausibly led to the apoptosis of lung cancer cells (Figure 3D). Aplasia Ras homolog member I (ARHI) inhibited the proliferation and caused arrest in the S phase of the cell cycle and led to apoptosis of SKOV3 ovarian cancer cells [40].

### 3.6. Knockout of TIPE2 Modulated Akt/mTOR/NF-κB Signaling Axis

The findings of our previous studies showed TIPE2 to have a profound role in the promotion of lung cancer cell proliferation, survival, and migration. Importantly, there are different signaling molecules or pathways associated with various hallmarks of cancer [42]. Therefore, it is imperative to decipher the associated signaling molecules/pathways to unravel the underlined molecular mechanism of action of TIPE2 in lung cancer cells. 

Our results showed that knockout of TIPE2 led to the downregulation of proteins involved in cell growth, survival, proliferation, and regulation of apoptosis, such as Cox-2, survivin, cIAP-1, XIAP, and Cyclin D1, and upregulated the expression of Caspase 9. Survivin, cIAP-1, and XIAP are the members of the inhibitor of apoptosis protein (IAP) family, which are associated with apoptosis inhibition [43]. Further, Caspase-9, a member of the Caspase family of cysteine proteases, is involved in cytokine processing and apoptosis [44]. Cox-2 is involved in the regulation of cellular growth, differentiation, and inflammation, whereas Cyclin D1 regulates cell cycle progression [43,45,46]. In addition, autophagy is a catabolic cellular mechanism in which degradation of cells’ dysfunctional components takes place through autophagosomes, which allows cell survival even under stress conditions, through maintenance of immune homeostasis. LC-3B is known as the most effective marker of autophagosome formation [47]. Notably, in TIPE2 knockout cells, downregulation of LC-3B was observed, which can be presumed to aid in reducing proliferation and survival of lung cancer cells. In addition, knockout of TIPE2 resulted in the downregulation of CXCR-4 and MMP-9, which are involved in the invasion, migration, and metastasis of cancer cells [48]. Further, the tumor suppressor protein ′p53′ controls different functions of the cells, such as regulation of apoptosis, senescence, reduction in cell growth, migration, and invasion. p21 is a target of p53, which is involved in inhibiting cell growth and reducing the invasive potential of tumor cells [49]. Notably, loss of TIPE2 expression efficiently resulted in the upregulation of these two tumor suppressor proteins.

**Figure 4:**
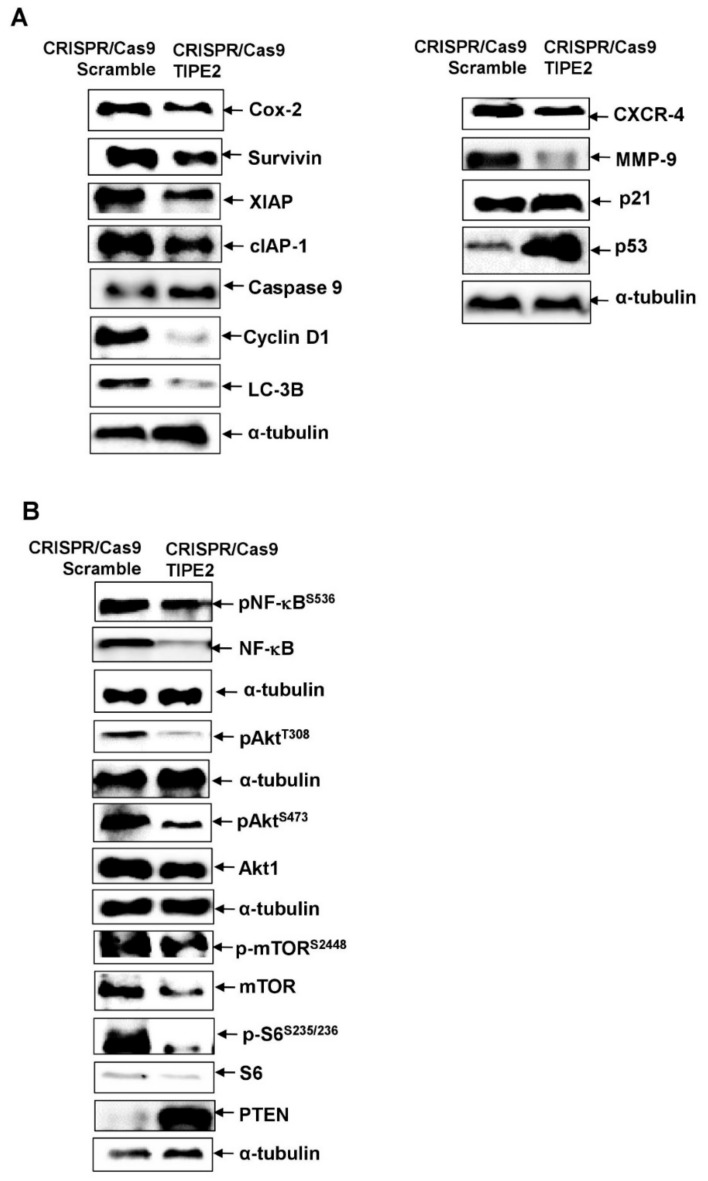
Effect of CRISPR/Cas9-mediated knockout of TIPE2 on different signaling molecules/pathways. (**A**) Effect of TIPE2 knockout on the expression of proteins involved in cell growth, proliferation, survival, and apoptosis regulation (Left panel); Effect of TIPE2 knockout on the expression of proteins involved in migration, metastasis, and effect on tumor suppressors p53 and p21 (Right panel). (**B**) Effect of TIPE2 knockout on Akt/mTOR/NF-κB signaling. α-tubulin was used as the loading control.

Mounting evidence imply that the PI3K/Akt/mTOR pathway plays a crucial role in oncogenesis and is often reported to be activated in lung cancer [5,6,50,51,52,53,54]. Aberrations in various messenger molecules of this pathway result in cancer cell proliferation, apoptosis inhibition, angiogenesis, and metastasis [55]. Therefore, we evaluated the association between this signaling axis and TIPE2-mediated lung cancer. Our findings revealed that knockout of TIPE2 caused downregulation of Akt1, p-Akt^S473^, p-Akt^T308^, mTOR, p-mTOR^S2448^, S6, and p-S6^S235/236^, the important constituents of the Akt/mTOR signaling cascade. In addition, phosphatase and tensin homolog (PTEN), a lipid and protein phosphatase, was found to be upregulated in TIPE2 knockout lung cancer cells. PTEN is a negative regulator of Akt and the loss of its function leads to the constitutive activation of Akt [55]. Thus, TIPE2 is observed to activate the Akt/mTOR signaling pathway, which might contribute to lung cancer pathogenesis. It is well evinced that constitutive activation of the PI3K/Akt leads to the aberrant NF-κB function [56,57]. Our results showed that knockout of TIPE2 effectively downregulated the expression of NF-κB and p-NF-κB^S536^. Hence, our findings clearly implied that knockout of TIPE2 led to decreased proliferation, survival, invasion, and migration of lung cancer cells by downregulating Akt/mTOR, S6, and NF-κB signaling and their downstream targets, which are involved in the regulation of different processes in lung cancer cells (Figure 4). 

### 3.7. Effect of Tobacco Components on the Proliferation of TIPE2 Knockout Lung Cancer Cells

**Figure 5:**
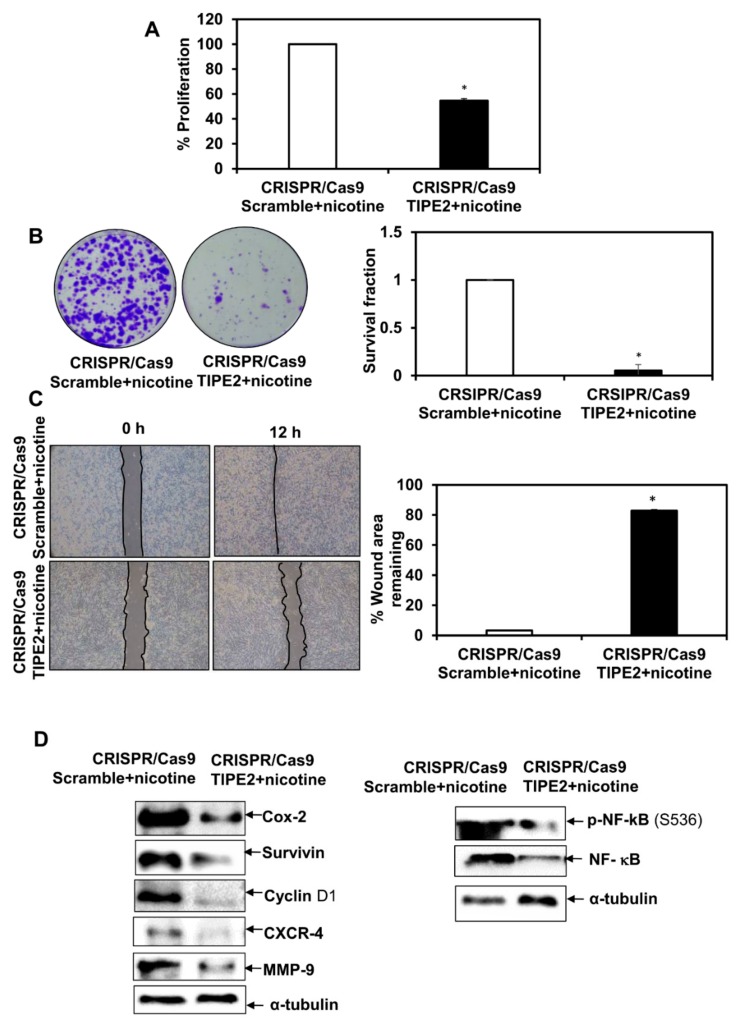
Effect of TIPE2 on nicotine-mediated lung carcinogenesis. (**A**) Effect of TIPE2 in nicotine-promoted proliferation of lung cancer cells evaluated using MTT assay. (**B**) Images of the colonies formed in nicotine-treated TIPE2 knockout cells along with the scrambled control (Left panel); graphical representation of clonogenic potential of nicotine-treated TIPE2 knockout cells in terms of survival fraction compared to scrambled control treated with nicotine. (**C**) Representative images showing the effect on the migration of nicotine-treated TIPE2 knockout cells along with the nicotine-treated scrambled control (Left panel); Graphical representation of the percent of the wound area remaining in nicotine-treated TIPE2 knockout cells compared to the nicotine-treated scrambled control (Right panel). (**D**) Effect on NF-κB and NF-κB-regulated gene products in nicotine-treated TIPE2 knockout cells. α-tubulin was used as the loading control. Data are represented as mean ± SE, * denotes *p* value < 0.05.

**Figure 6:**
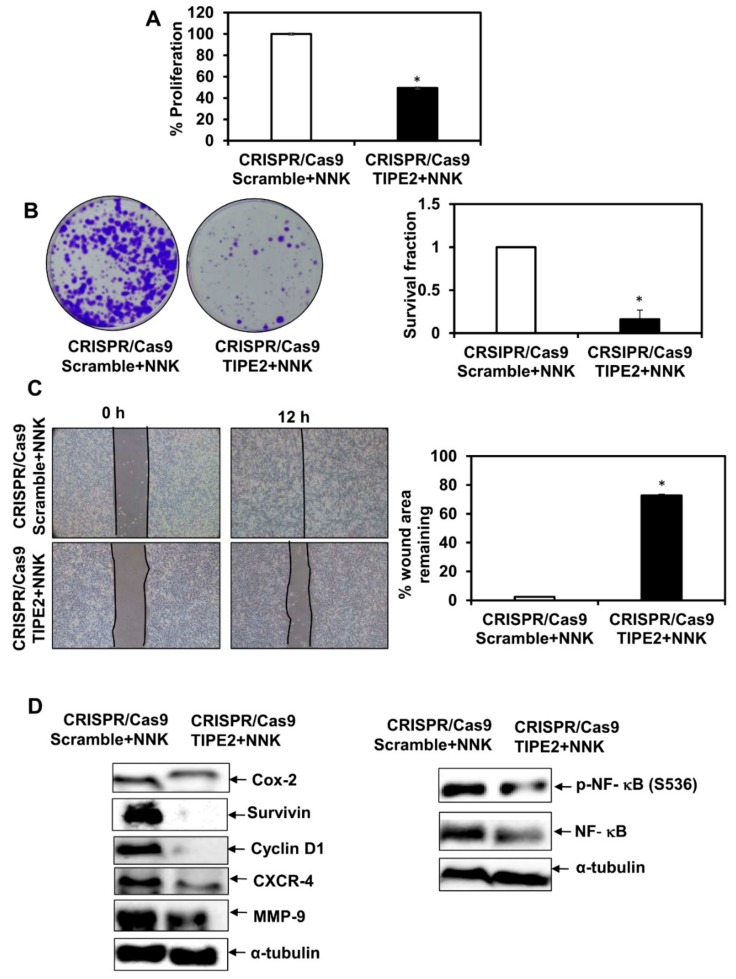
Effect of TIPE2 on NNK-mediated lung carcinogenesis. (**A**) Effect of TIPE2 in NNK-promoted proliferation of lung cancer cells evaluated via MTT assay. (**B**) Images of the colonies formed in NNK-treated TIPE2 knockout cells (Left panel); graphical representation of clonogenic potential of NNK-treated TIPE2 knockout cells in terms of survival fraction compared to scrambled control cells treated with NNK. (**C**) Representative images showing the effect on the migration of NNK-treated TIPE2 knockout cells along with the NNK-treated scrambled control (Left panel); Graphical representation of the percent of the wound area remaining in NNK-treated TIPE2 knockout cells compared to the NNK-treated scrambled control (Right panel). (**D**) Effect on NF-κB and NF-κB-regulated gene products in NNK-treated TIPE2 knockout cells. α-tubulin was used as the loading control. Data are represented as mean ± SE, * denotes *p* value < 0.05.

**Figure 7:**
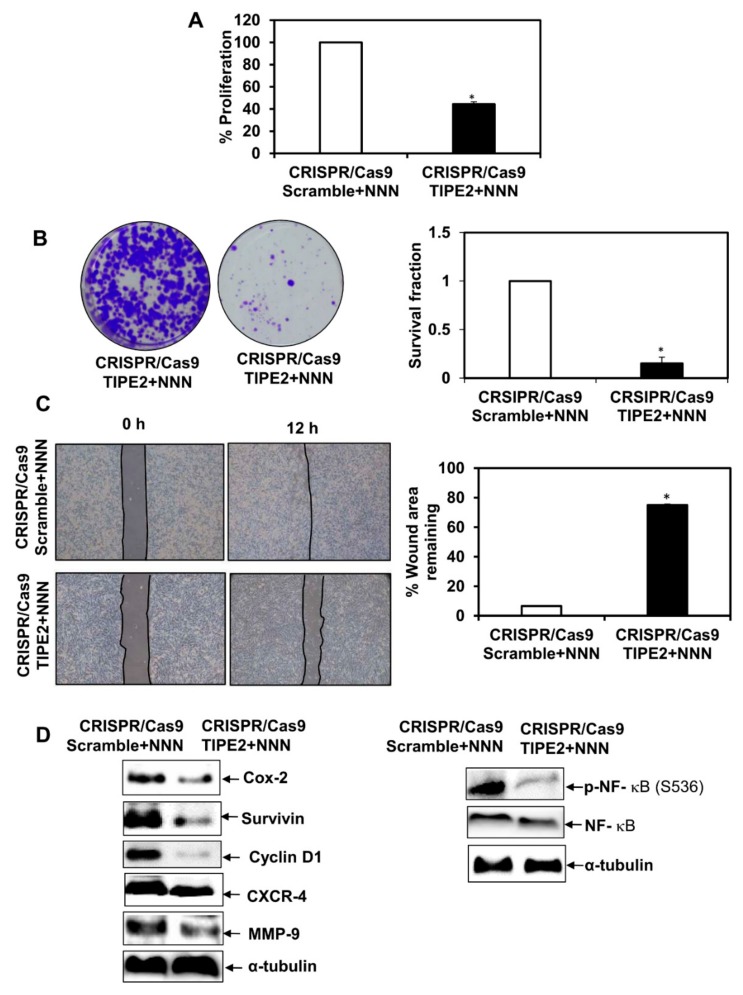
Effect of TIPE2 on NNN-mediated lung carcinogenesis. (**A**) Effect of TIPE2 in NNN-promoted proliferation of lung cancer cells evaluated via MTT assay. (**B**) Images of the colonies formed in NNN-treated TIPE2 knockout cells (Left panel); graphical representation of clonogenic potential of NNN-treated TIPE2 knockout cells in terms of survival fraction compared to scrambled control cells treated with NNN. (**C**) Representative images showing the effect on the migration of NNN-treated TIPE2 knockout cells along with the NNN-treated scrambled control (Left panel); Graphical representation of the percent of the wound area remaining in NNN-treated TIPE2 knockout cells compared to the NNN-treated scrambled control (Right panel). (**D**) Effect on NF-κB and NF-κB-regulated gene products in NNN-treated TIPE2 knockout cells. α-tubulin was used as the loading control. Data are represented as mean ± SE, * denotes *p* value < 0.05.

**Figure 8:**
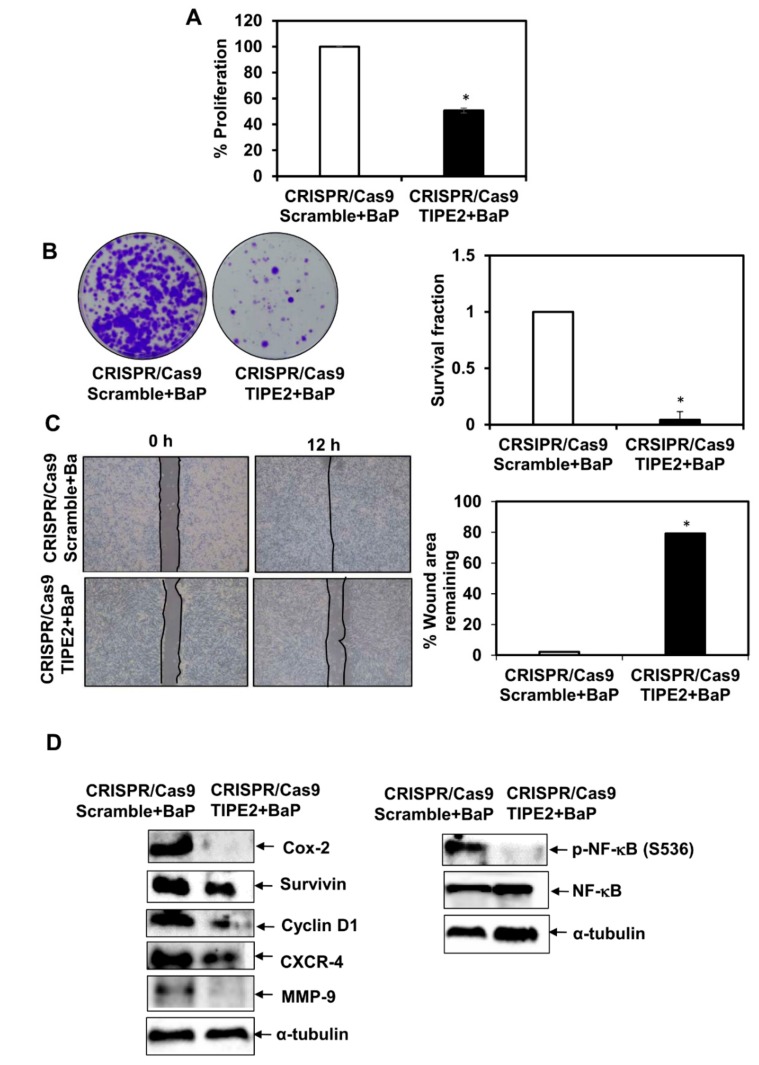
Effect of TIPE2 on BaP-induced lung carcinogenesis. (**A**) Effect of TIPE2 in BaP-promoted proliferation of lung cancer cells evaluated via MTT assay. (**B**) Images of the colonies formed in BaP-treated TIPE2 knockout cells (Left panel); graphical representation of clonogenic potential of BaP-treated TIPE2 knockout cells in terms of survival fraction compared to scrambled control cells treated with BaP. (**C**) Representative images showing the effect on the migration of BaP-treated TIPE2 knockout cells along with BaP-treated scrambled control (Left panel); Graphical representation of the percent of the wound area remaining in BaP-treated TIPE2 knockout cells compared to the BaP-treated scrambled control (Right panel). (**D**) Effect on NF-κB and NF-κB-regulated gene products in BaP-treated TIPE2 knockout cells. α-tubulin was used as the loading control. Data are represented as mean ± SE, * denotes *p* value < 0.05.

In order to see the effect of nicotine, NNK, NNN, and BaP on the proliferation of NCIH460 human lung cancer cells after knockout TIPE2, the MTT assay was performed (Figure 5A, Figure 6A, Figure 7A and Figure 8A). Upon treatment with all four tobacco components separately, a decrease in the proliferation of TIPE2 knockout cells compared to the scrambled control cells treated with the respective components was observed. Thus, these results indicate TIPE2 to be involved in the positive regulation of tobacco-promoted proliferation of lung cancer cells. 

### 3.8. Effect of Tobacco Components on the Clonogenic Potential of TIPE2 Knockout Lung Cancer Cells

For determining the effect of nicotine, NNK, NNN, and BaP on the clonogenic potential of NCIH460 lung cancer cells after knockout of TIPE2, a colony formation assay was performed (Figure 5B, Figure 6B, Figure 7B and Figure 8B). Similar to the proliferation assay, in the colony formation assay, decreased survival fractions of TIPE2 knockout cells treated with nicotine, NNK, NNN, and BaP were observed compared to scrambled control cells treated with the same. These results suggested that knockout of TIPE2 reduced tobacco components’ promoted survival of lung cancer cells. 

### 3.9. Effect of Tobacco Components on the Migration Potential of TIPE2 Knockout Lung Cancer Cells

Tobacco and its components are well evinced to have significant association with the migration of lung cancer cells. To determine the migration potential of tobacco components’-treated TIPE2 knockout cells, a wound healing assay was performed. The results showed that in the case of tobacco components’-treated scrambled control cells, there was complete healing of the wound at 12 h, whereas the untreated scrambled control, as shown earlier, took 24 h for the healing of the wound, which indicates the ability of these components to increase the migration as well as invasive potential of the lung cancer cells (Figure 5C, Figure 6C, Figure 7C and Figure 8C). In the case of nicotine, NNK, NNN, and BaP treatment, TIPE2 knockout cells exerted significant inhibition in the wound healing. Thus, these results clearly suggested TIPE2 to be involved in the positive regulation of tobacco-promoted migration of lung cancer cells.

### 3.10. Effect of Tobacco Components on the Modulation of NF-κB and NF-κB-Regulated Gene Products in TIPE2 Knockout Lung Cancer Cells

Tobacco-promoted lung carcinogenesis is driven by alterations in different signal transduction cascades. From the above findings, we observed TIPE2 to be involved in the positive regulation of nicotine-, NNK-, NNN-, and BaP-mediated proliferation, survival, and migration of lung cancer cells. Therefore, we determined the molecular targets which are involved in the TIPE2-mediated tumorigenic effect in tobacco facilitated lung cancer. In NNK treated TIPE2 knockout cells, downregulation in the expression of proteins involved in cell proliferation, growth, survival, invasion, migration, and metastasis, such as Cox-2, Cyclin D1, survivin, CXCR-4, and MMP-9, was observed. In addition, NNK-treated TIPE2 knockout cells showed downregulation in both NF-κB and p-NF-κB^S536^. Similarly, in the case of nicotine-, NNN-, and BaP-treated TIPE2 knockout cells, downregulation of Cox-2, Cyclin D1, survivin, CXCR-4, and MMP-9 was observed. In addition, nicotine-, NNN-, and BaP-treated TIPE2 knockout cells exerted downregulation in p-NF-κB^S536^ compared to the scrambled control cells treated with the respective compounds. Taken together, TIPE2 is involved in the positive regulation of tobacco-mediated lung carcinogenesis via enhanced proliferation, survival, and migration through inflection of NF-κB and NF-κB-regulated gene products which are responsible for growth, proliferation, survival, invasion, migration, and metastasis of lung cancer cells (Figure 5D, Figure 6D, Figure 7D and Figure 8D).

## 4. Discussion

Lung cancer is an extremely invasive neoplasm with a poor survival rate. Notably, high mortality due to lung cancer can be attributed to its susceptibility for early spread, lack of effective biomarkers for early diagnosis and prognosis and late stage detection [58]. Increasing lines of evidence suggest TIPE2, a newly identified protein, to play pivotal role in the modulation of tumorigenesis, inflammation, cell death, and other cellular activities. It has been found to be strongly associated with different cancers and several chronic diseases [19,59]. Therefore, the present study focused on deciphering the role of this protein in the pathogenesis of lung cancer, which would certainly help us to develop effective biomarkers and targets for the management of this cancer type.

Initially, we analyzed the expression of TIPE2 in human lung cancer tissues. Our analysis revealed TIPE2 to be significantly upregulated in lung cancer tissues when compared to normal lung tissues. TIPE2, a negative regulator of innate as well as cellular immunity, was reported to be significantly downregulated in human breast cancer cells as well as tissues, and its overexpression inhibited the proliferation of tumor cells and tumor growth [22]. Its expression was found to be remarkably less in glioma cells [23]. Further, in a study conducted in NSCLC, TIPE2 displayed a higher expression level in normal lung tissues compared to the NSCLC tissues. Besides, downregulation of TIPE2 was found to be well-correlated with advanced TNM stage [60]. In addition, a study conducted by Liu and colleagues evaluated the expression of TIPE2 in the tissues of different lung cancer pathologies, such as lung squamous cancer, small cell lung cancer, and lung adenocarcinoma. The findings showed that TIPE2 expression was lost in small cell lung cancer in comparison with the nearby non-malignant tissues. Its overexpression led to the inhibition of the growth of lung cancer cells in vitro and tumor formation in vivo [61]. Contrary to the above-mentioned findings, we found that this protein was significantly upregulated in malignant lung tissues compared to the normal lung tissues. A similar observation was reported by Hao and colleagues, where enhanced expression of TIPE2 was found in both diffuse large B-cell lymphoma and peripheral T-cell lymphoma [62]. In line with our findings, a study carried out by Li and their group showed TIPE2 to be upregulated in NSCLC tumor tissues when compared with adjacent normal tissues [28]. Further, in our study, TIPE2 showed upregulation in different pathologies of lung cancer, such as adenocarcinoma, squamous cell carcinoma, adenosquamous carcinoma, and large cell carcinoma compared to normal lung tissues. Thus, TIPE2 plays an important role in lung carcinogenesis, though it was observed to exert site-specific expression. In addition, we found TIPE2 to be significantly upregulated in different stages and grades of lung tumor. However, contrary to our findings, overexpression of TIPE2 was reported to be negatively correlated with the advanced clinical stage of lung cancer [28].

Further, we observed TIPE2 to be involved in inducing proliferation, survival, and migration of lung cancer cells. In contrast to our findings, TIPE2 was reported to block the proliferation, migration, invasion, and *in vivo* tumorigenesis in the case of breast cancer through the involvement of the Akt and p38 pathways [63]. In the case of another study, TIPE2 overexpression was reported to notably inhibit the proliferation of breast cancer cells. They showed that hydrodynamic gene delivery of TIPE2 plasmids *in vivo* resulted in the marked inhibition of breast cancer cells’ growth and metastasis. Further, it increased the production of interferon gamma (IFN-γ) and tumor necrosis factor alpha (TNF-α) by CD8^+^ T and NK cells in spleens as well as tumor microenvironment and increased the cytotoxic effects of CD8^+^ T and NK cells [64]. It inhibited the migration and invasion of breast cancer cells via EMT. Further, TIPE2 was found to downregulate the expression of different proteins, such as β-catenin, cyclin D1, and c-Myc in breast cancer cells [22]. In addition, overexpression of TIPE2 led to the inhibition of hypoxia-induced migration as well as invasion and EMT in glioma cells. Mechanistically, TIPE2 overexpression blocked β-catenin, cyclin D1, and c-myc expression induced by hypoxia [23]. Further, it suppressed the metastasis of gastric cancer cells through downregulation of the β-catenin signaling pathway [24]. In addition, in a study conducted by Li and their group, overexpression of TIPE2 was reported to inhibit the colony forming ability of human NSCLC cells, whereas our results showed the opposite effect [28]. Again, contrary to our results, its overexpression caused inhibition in the migration of lung and prostate cancer cells [28,29]. It was reported to suppress tumor invasiveness and angiogenesis through blockage of Rac1 and its downstream mediators, which include F-actin polymerization and vascular endothelial growth factor (VEGF) [28]. Further, TIPE2 promoted lung cancer cell apoptosis via modulation of caspase-3, -9, Bcl-2, and Bax through the P38 and Akt pathways [61]. In addition, overexpression of TIPE2 inhibited the proliferation, colony formation, invasion, and the expression of Bcl-XL and N-cadherin, involved in regulating apoptosis and EMT phenomenon in lung cancer cells [60].

**Figure 9:**
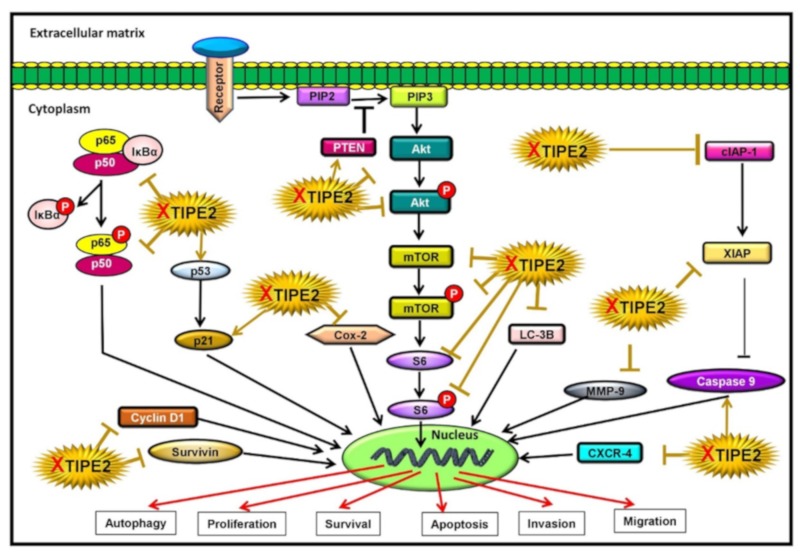
Knockout of TIPE2 modulates Akt/mTOR/NF-κB signaling, ‘XTIPE2′ denotes CRISPR/Cas9 TIPE2.

Besides, we found that knockout of TIPE2 led to the increased cell number in the S phase of the cell cycle in lung cancer cells. The arrest in the S phase as a result of TIPE2 knockout might induce apoptosis of lung cancer cells. In a study conducted in human ovarian cancer, Aplasia Ras homolog member I (ARHI) was reported to inhibit the proliferation as well as cause S phase cell cycle arrest and result in the apoptosis of SKOV3 cells [40]. Further, treatment with pemetrexed, the first line therapy approved for metastatic or advanced NSCLC, led to the arrest in the S phase of the cell cycle and induction of apoptosis via deregulated activation of the Akt pathway in A549 lung cancer cells [65]. However, it needs to be further elucidated whether the DNA histogram (Figure 3C) indicates S phase arrest or nuclei with decreased DNA contents after G2 apoptosis are interpreted in S phase arrest. In this study, we also found that knockout of TIPE2 resulted in the downregulation of Akt/mTOR, S6, and NF-κB signaling and their downstream targets, which are involved in diverse cellular processes linked with lung cancer. The involvement of Akt in TIPE2-mediated carcinogenesis was also reported in the case of gastric and prostate cancers. However, in those studies, TIPE2 exerted the opposite effect of that obtained in our study. For instance, overexpression of TIPE2 suppressed the growth of gastric cancer cells via reduction of Akt and ERK1/2 [26]. Similarly, Wu and their group also showed that TIPE2 suppressed the metastasis of gastric cancer cells via Akt inhibition [24]. In prostate cancer cells, upregulation of TIPE2 inhibited the migration, invasion, and EMT through blockage of the PI3K/Akt signaling [29]. Additionally, a study on HCC showed NF-κB to be involved in TIPE2 mediated lung cancer. TIPE2 suppressed TNF-α induced metastasis of HCC cells via inhibition of NF-κB and ERK1/2 [27]. In addition, TIPE2 also exerted its function through regulation of autophagy, as LC-3B was downregulated in TIPE2 knockout cells. Further, modulation in the expression of p53 and p21 by TIPE2 also signifies its role in regulating various hallmarks of cancer (Figure 9).

Tobacco-carcinogen-transformed human bronchial epithelial cells have been found to exert enhanced PI3K/Akt activation, leading to their increased proliferation and survival [66]. Furthermore, the binding of potent lung carcinogens, NNK and NNN, to the nAChR, induces proliferation, survival, migration, and invasion of tumor cells [67]. In addition, nicotine is also known to induce proliferation and angiogenesis in different cellular models [68]. Further, BaP was also reported to effectively promote the cell proliferation in lung cancer [69]. Additionally, tobacco components are well known to have significant association with cancer cell migration. Chronic exposure to cigarette smoke led to the activation of PAK6 in NSCLC, which regulates different processes in cancer cells [70]. Further, components present in tobacco such as nicotine and NNK are also reported to influence the migration potential of lung cancer cells [71,72]. Therefore, we evaluated the effect of nicotine, NNK, NNN, and BaP on the proliferation, survival, and migration of NCIH460 human lung cancer cells after knockout of TIPE2. Upon treatment with all four tobacco components, there was a decrease in the proliferation of TIPE2 knockout cells. Similar to the proliferation assay, in the colony formation assay as well, decreased survival fractions of TIPE2 knockout cells treated with nicotine, NNK, NNN, and BaP were observed, as denoted by the decreased number of colonies compared to scrambled control cells treated with the same. Further, nicotine-, NNK-, NNN-, and BaP-treated TIPE2 knockout cells exerted decreased migration potential as well. Thus, these results clearly suggested TIPE2 to be strongly involved in mediating tobacco-promoted proliferation, survival, and migration of lung cancer cells.

As mentioned before, tobacco-mediated lung carcinogenesis is driven by variations in different signaling pathways. Hence, we identified those molecular targets through which TIPE2 might mediate its tumorigenic effect in tobacco-facilitated lung cancer. TIPE2 is found to be involved in the positive regulation of tobacco-promoted lung carcinogenesis by modulating the expression of NF-κB and NF-κB-regulated gene products, such as Cox-2, survivin, Cyclin D1, MMP-9, and CXCR-4. NF-κB is a transcription factor that regulates the expression of genes involved in lung carcinogenesis [73,74,75,76,77,78,79,80,81,82,83]. Alvira and their group reported high levels of nuclear NF-κB in lung cancer tissues and enhanced NF-κB activity well-correlated with more advanced disease in lung adenocarcinoma [84]. Further, exposure to NNK was reported to activate NF-κB, which subsequently enhanced the expression of Cyclin D1 and helped in the proliferation of normal human bronchial epithelial as well as small airway epithelial cells [73]. Additionally, cigarette smoke and its components, such as nicotine and NNK are reported to activate NF-κB in various NSCLC cells [85]. Altogether, we found TIPE2 to play a vital role in tobacco-promoted lung cancer and therefore, specific targeting of it holds enormous prospect in newer therapeutic interventions in lung cancer.

## 5. Conclusions

This study shows TIPE2 to be involved in the positive regulation of lung cancer. Further, this is the first report which shows the correlation between tobacco constituents and the regulation of TIPE2 in human lung cancer. In addition, knockout of TIPE2 reduced the proliferation, survival, invasion, and migration of human lung cancer cells. Further, mechanistic studies revealed that TIPE2 exerted its effect through modulation of the Akt/mTOR/NF-κB signaling axis. In addition, our results showed for the first time that TIPE2 is involved in the positive regulation of nicotine-, NNK-, NNN-, and BaP-promoted proliferation, survival, and migration of lung cancer cells through modulation of NF-κB and NF-κB-regulated gene products. Taken together, TIPE2 plays a critical role in the development and progression of lung cancer and also in tobacco-promoted lung carcinogenesis. Thus, targeting this protein holds an enormous prospect in therapeutic interventions for the effective management of lung cancer. Nevertheless, these findings need to be further validated in the *in vivo* and clinical settings to fully establish the diagnostic and prognostic significance of this newly identified important protein.

## Supplementary Materials

The following are available online at https://www.mdpi.com/2218-273X/9/12/836/s1, Table S1: Lung cancer tissue array details, Table S2: sgRNA target sequences, Table S3: Primary and secondary antibodies used for Western blot analysis.
