# Supplementary material for: TIPE2 Induced the Proliferation, Survival, and Migration of Lung Cancer Cells Through Modulation of Akt/mTOR/NF-κB Signaling Cascade
Source: Biomolecules. 2019 Dec 6;9(12):836. doi: 10.3390/biom9120836 (PMC6995575; doi:10.3390/biom9120836)
Supplement: Supplementary file 1 [file biomolecules-09-00836-s001.pdf]

Table S1: Lung cancer tissue array details

Position	Age	Sex	Organ	Pathology diagnosis	TNM	Grade	Stage	Type
A1	68	M	Lung	Adenocarcinoma	T2N0M0	2-3	I	Malignant
A2	37	M	Lung	Adenocarcinoma	T2N0M0	2	I	Malignant
A3	47	M	Lung	Adenocarcinoma	T1N0M0	2-3	I	Malignant
A4	46	M	Lung	Adenocarcinoma	T1N0M0	2	I	Malignant
A5	67	M	Lung	Adenocarcinoma	T2N0M0	2	I	Malignant
A6	66	M	Lung	Adenocarcinoma	T1N0M0	2	I	Malignant
A7	70	M	Lung	Adenocarcinoma	T2N0M0	2	I	Malignant
A8	59	M	Lung	Adenocarcinoma	T2N1M0	2-3	II	Malignant
A9	60	F	Lung	Adenocarcinoma (sparse)	T2N0M0	-	I	Malignant
A10	58	M	Lung	Adenocarcinoma	T2N1M0	2	II	Malignant
A11	70	M	Lung	Adenocarcinoma	T1N0M0	2	I	Malignant
A12	52	F	Lung	Adenocarcinoma	T2N0M0	2	I	Malignant
A13	70	M	Lung	Adenocarcinoma	T1N0M0	2	I	Malignant
A14	67	F	Lung	Adenocarcinoma	T2N0M0	2	I	Malignant
A15	45	M	Lung	Adenocarcinoma	T2N0M0	2	I	Malignant
B1	68	M	Lung	Adenocarcinoma	T2N0M0	2-3	I	Malignant
B2	37	M	Lung	Adenocarcinoma	T2N0M0	2	I	Malignant
B3	47	M	Lung	Adenocarcinoma	T1N0M0	2-3	I	Malignant
B4	46	M	Lung	Adenocarcinoma	T1N0M0	2	I	Malignant
B5	67	M	Lung	Adenocarcinoma	T2N0M0	2	I	Malignant
B6	66	M	Lung	Adenocarcinoma	T1N0M0	2	I	Malignant
B7	70	M	Lung	Adenocarcinoma	T2N0M0	2	I	Malignant
B8	59	M	Lung	Adenocarcinoma	T2N1M0	2-3	II	Malignant
B9	60	F	Lung	Adenocarcinoma (fibrous tissue and blood vessel)	T2N0M0	-	I	Malignant
B10	58	M	Lung	Adenocarcinoma	T2N1M0	2	II	Malignant
B11	70	M	Lung	Adenocarcinoma	T1N0M0	2	I	Malignant
B12	52	F	Lung	Adenocarcinoma	T2N0M0	2	I	Malignant
B13	70	M	Lung	Adenocarcinoma	T1N0M0	2	I	Malignant
B14	67	F	Lung	Adenocarcinoma	T2N0M0	2	I	Malignant
B15	45	M	Lung	Adenocarcinoma	T2N0M0	2	I	Malignant
C1	62	M	Lung	Adenocarcinoma	T2N0M0	2	I	Malignant
C2	60	M	Lung	Adenocarcinoma	T2N0M0	2	I	Malignant
C3	62	F	Lung	Mucinous adenocarcinoma (sparse)	T2N0M0	1	I	Malignant

C4	51	M	Lung	Adenocarcinoma	T2N0M0	3	I	Malignant
C5	37	F	Lung	Adenocarcinoma	T2N0M0	2	I	Malignant
C6	66	F	Lung	Adenocarcinoma	T2N1M0	2	II	Malignant
C7	67	F	Lung	Adenocarcinoma	T2N0M0	2	I	Malignant
C8	50	F	Lung	Adenocarcinoma	T3N0M0	3	IIIa	Malignant
C9	54	F	Lung	Adenocarcinoma with necrosis (sparse)	T3N0M0	3	IIIa	Malignant
C10	49	F	Lung	Adenocarcinoma	T2N1M0	3	II	Malignant
C11	51	F	Lung	Adenocarcinoma	T2N0M0	3	I	Malignant
C12	61	M	Lung	Adenocarcinoma	T2N0M0	3	I	Malignant
C13	65	M	Lung	Adenocarcinoma	T2N0M0	3	I	Malignant
C14	61	M	Lung	Adenocarcinoma	T1N0M0	3	I	Malignant
C15	67	F	Lung	Adenosquamous carcinoma	T2N1M0	-	II	Malignant
D1	62	M	Lung	Adenocarcinoma	T2N0M0	2	I	Malignant
D2	60	M	Lung	Adenocarcinoma	T2N0M0	2	I	Malignant
D3	62	F	Lung	Mucinous adenocarcinoma	T2N0M0	1	I	Malignant
D4	51	M	Lung	Adenocarcinoma	T2N0M0	3	I	Malignant
D5	37	F	Lung	Adenocarcinoma	T2N0M0	2	I	Malignant
D6	66	F	Lung	Adenocarcinoma	T2N1M0	2	II	Malignant
D7	67	F	Lung	Adenocarcinoma with necrosis	T2N0M0	2	I	Malignant
D8	50	F	Lung	Adenocarcinoma	T3N0M0	3	IIIa	Malignant
D9	54	F	Lung	Adenocarcinoma	T3N0M0	3	IIIa	Malignant
D10	49	F	Lung	Adenocarcinoma	T2N1M0	3	II	Malignant
D11	51	F	Lung	Adenocarcinoma	T2N0M0	3	I	Malignant
D12	61	M	Lung	Adenocarcinoma	T2N0M0	3	I	Malignant
D13	65	M	Lung	Adenocarcinoma	T2N0M0	3	I	Malignant
D14	61	M	Lung	Adenocarcinoma	T1N0M0	3	I	Malignant
D15	67	F	Lung	Adenosquamous carcinoma	T2N1M0	-	II	Malignant
E1	43	M	Lung	Adenosquamous carcinoma	T2N0M0	-	I	Malignant
E2	70	M	Lung	Adenosquamous carcinoma	T2N0M0	-	I	Malignant
E3	54	M	Lung	Squamous cell carcinoma	T2N3M0	1	IIIa	Malignant
E4	56	M	Lung	Squamous cell carcinoma	T3N1M0	1	IIIa	Malignant
E5	56	M	Lung	Squamous cell carcinoma	T3N0M0	1	IIIa	Malignant
E6	59	M	Lung	Squamous cell carcinoma	T2N1M0	2	II	Malignant

E7	67	M	Lung	Squamous cell carcinoma	T1N0M0	2	I	Malignant
E8	50	M	Lung	Squamous cell carcinoma (tumoral necrosis)	T2N0M0	-	I	Malignant
E9	53	M	Lung	Squamous cell carcinoma with necrosis	T2N0M0	3	I	Malignant
E10	61	M	Lung	Squamous cell carcinoma	T2N0M0	3	II	Malignant
E11	71	F	Lung	Squamous cell carcinoma	T2N0M0	3	I	Malignant
E12	56	M	Lung	Squamous cell carcinoma	T2N1M0	1	I	Malignant
E13	70	F	Lung	Squamous cell carcinoma with necrosis	T2N0M0	2	II	Malignant
E14	48	M	Lung	Squamous cell carcinoma	T2N0M0	2	I	Malignant
E15	44	M	Lung	Squamous cell carcinoma	T2N0M0	2	I	Malignant
F1	43	M	Lung	Adenosquamous carcinoma (carcinoma sparse)	T2N0M0	-	I	Malignant
F2	70	M	Lung	Adenosquamous carcinoma with necrosis (sparse)	T2N3M0	1	I	Malignant
F3	54	M	Lung	Squamous cell carcinoma	T3N1M	1	IIIa	Malignant
F4	56		Lung	Squamous cell carcinoma	T3N0M	1	IIIa	Malignant
F5	56	M	Lung	Squamous cell carcinoma	T2N1M0	2	IIIa	Malignant
F6	59	M	Lung	Squamous cell carcinoma	T1N0M0	2	II	Malignant
F7	67	M	Lung	Squamous cell carcinoma	T2N0M0	-	I	Malignant
F8	50	M	Lung	Squamous cell carcinoma with necrosis (sparse)	T2N0M0	3	I	Malignant
F9	53	M	Lung	Squamous cell carcinoma with necrosis	T2N1M0	3	I	Malignant
F10	61	M	Lung	Squamous cell carcinoma	T2N0M0	3	II	Malignant
F11	71	F	Lung	Squamous cell carcinoma	T2N0M0	1	I	Malignant

F12	56	M	Lung	Squamous cell carcinoma	T2N1M0	2	I	Malignant
F13	70	F	Lung	Squamous cell carcinoma with necrosis	T2N0M0	2	II	Malignant
F14	48	M	Lung	Squamous cell carcinoma	T2N0M0	2	I	Malignant
F15	44	M	Lung	Squamous cell carcinoma	T3N1M0	-	I	Malignant
G1	50	M	Lung	Squamous cell carcinoma	T2N1M0	2	IIIa	Malignant
G2	57	M	Lung	Squamous cell carcinoma	T2N1M0	2	II	Malignant
G3	65	M	Lung	Squamous cell carcinoma	T2N0M0	1	II	Malignant
G4	60	M	Lung	Squamous cell carcinoma (interstitial pneumonia)	T2N0M0	-	I	Malignant
G5	65	M	Lung	Squamous cell carcinoma	T3N0M0	1	IIIa	Malignant
G6	60	M	Lung	Squamous cell carcinoma	T2N0M0	2	I	Malignant
G7	54	F	Lung	Squamous cell carcinoma	T2N1M0	3	II	Malignant
G8	61	F	Lung	Squamous cell carcinoma	T2N0M0	2	I	Malignant
G9	61	M	Lung	Squamous cell carcinoma	T2N0M0	2	I	Malignant
G10	71	M	Lung	Squamous cell carcinoma	T2N1M0	3	II	Malignant
G11	49	F	Lung	Squamous cell carcinoma	T2N0M0	3	I	Malignant
G12	69	M	Lung	Squamous cell carcinoma	T2N0M0	2	I	Malignant
G13	49	M	Lung	Squamous cell carcinoma	T2N0M0	2	I	Malignant
G14	55	F	Lung	Squamous cell carcinoma	T2N0M0	3	I	Malignant
G15	65	M	Lung	Squamous cell carcinoma	T2N0M0	3	I	Malignant
H1	50	M	Lung	Squamous cell carcinoma with necrosis	T2N1M0	2	IIIa	Malignant
H2	57	M	Lung	Squamous cell carcinoma (fibrous tissue and blood vessel)	T2N1M0	2	II	Malignant
H3	65	M	Lung	Squamous cell carcinoma	T2N0M0	1	II	Malignant

H4	60	M	Lung	Squamous cell carcinoma (interstitial pneumonia)	T2N0M0	-	I	Malignant
H5	65	M	Lung	Squamous cell carcinoma	T3N0M0	1	IIIa	Malignant
H6	60	M	Lung	Squamous cell carcinoma	T2N0M0	2	I	Malignant
H7	54	F	Lung	Squamous cell carcinoma	T2N1M0	3	II	Malignant
H8	61	F	Lung	Squamous cell carcinoma	T2N0M0	2	I	Malignant
H9	61	M	Lung	Squamous cell carcinoma	T2N0M0	2	I	Malignant
H10	71	M	Lung	Squamous cell carcinoma	T2N1M0	3	II	Malignant
H11	49	F	Lung	Squamous cell carcinoma	T2N0M0	3	I	Malignant
H12	69	M	Lung	Squamous cell carcinoma	T2N0M0	2	I	Malignant
H13	49	M	Lung	Squamous cell carcinoma	T2N0M0	2	I	Malignant
H14	55	F	Lung	Squamous cell carcinoma	T2N0M0	3	I	Malignant
H15	65	M	Lung	Squamous cell carcinoma	T2N0M0	3	I	Malignant
I1	68	M	Lung	Squamous cell carcinoma	T3N0M0	3	IIIa	Malignant
I2	46	M	Lung	Bronchioalveolar carcinoma	T2N0M0	-	I	Malignant
I3	55	F	Lung	Bronchioalveolar carcinoma	T2N0M0	-	I	Malignant
I4	54	M	Lung	Small cell undifferentiated carcinoma	T2N1M0	-	II	Malignant
I5	73	M	Lung	Small cell undifferentiated carcinoma with necrosis	T3N1M0	-	IIIa	Malignant
I6	66	F	Lung	Small cell undifferentiated carcinoma	T2N1M0	-	II	Malignant
I7	51	F	Lung	Small cell undifferentiated carcinoma with necrosis	T1N0M0	-	I	Malignant
I8	62	M	Lung	Large cell carcinoma	T3N0M0	-	IIIa	Malignant
I9	57	M	Lung	Large cell carcinoma	T3N0M0	-	IIIa	Malignant

I10	64	M	Lung	Neuroendocrine carcinoma	T2N2M0	-	IIIa	Malignant
I11	22	M	Lung	Normal lung tissue	-	-	-	Normal
I12	25	M	Lung	Normal lung tissue	-	-	-	Normal
I13	46	M	Lung	Normal lung tissue	-	-	-	Normal
I14	41	F	Lung	Normal lung tissue	-	-	-	Normal
I15	40	F	Lung	Normal lung tissue	-	-	-	Normal
J1	68	M	Lung	Squamous cell carcinoma	T3N0M0	3	IIIa	Malignant
J2	46	M	Lung	Bronchioalveolar carcinoma with necrosis	T2N0M0	-	I	Malignant
J3	55	F	Lung	Bronchioalveolar carcinoma	T2N0M0	-	I	Malignant
J4	54	M	Lung	Small cell undifferentiated carcinoma	T2N1M0	-	II	Malignant
J5	73	M	Lung	Small cell undifferentiated carcinoma with necrosis	T3N1M0	-	IIIa	Malignant
J6	66	F	Lung	Small cell un-differentiated carcinoma	T2N1M0	-	II	Malignant
J7	51	F	Lung	Small cell undifferentiated carcinoma with necrosis	T1N0M0	-	I	Malignant
J8	62	M	Lung	Large cell carcinoma	T3N0M0	-	IIIa	Malignant
J9	57	M	Lung	Large cell carcinoma	T3N0M0	-	IIIa	Malignant
J10	64	M	Lung	Neuroendocrine carcinoma	T2N2M0	-	IIIa	Malignant
J11	22	M	Lung	Normal lung tissue	-	-	-	Normal
J12	25	M	Lung	Normal lung tissue	-	-	-	Normal
J13	46	M	Lung	Normal lung tissue	-	-	-	Normal
J14	41	F	Lung	Normal lung tissue	-	-	-	Normal
J15	40	F	Lung	Normal lung tissue	-	-	-	Normal

Table S2: sgRNA target sequences

Gene	Target	sgRNA target sequence
Scramble	-	GCACTCACATCGCTACATCA
TNFAIP8L2 (TIPE2)	Target 1	CCAAGGAGTACACGCACAGC
	Target 2	CAGGTCCTTGATCACGCGCT
	Target 3	CCCGCTTTCGCCAGAAGCTG

Table S3: Primary and secondary antibodies used for Western blot analysis

Name	Details	Dilutions used
Anti-TNFAIP8L2 (TIPE2) antibody	ab110389; abcam®, Cambridge, USA	1:4000
Anti-α-Tubulin antibody	2144S; Cell Signaling Technology, Massachusetts, USA	1:2000
Anti-Phospho- Akt (Ser473) antibody	4060S; Cell Signaling Technology, Massachusetts, USA	1: 4000
Anti-Phospho- Akt (Thr308) antibody	244F9; Cell Signaling Technology, Massachusetts, USA	1:2000
Anti- Akt1 antibody	2938S; Cell Signaling Technology, Massachusetts, USA	1: 2000
Anti-Phospho- mTOR protein (Ser2448) antibody	5536T; Cell Signaling Technology, Massachusetts, USA	1: 2000
Anti-mTOR antibody	2983T; Cell Signaling Technology, Massachusetts, USA	1: 2000
Anti-Phospho- S6 Ribosomal protein (Ser235/236) antibody	4858T; Cell Signaling Technology, Massachusetts, USA	1: 2000
Anti-S6 Ribosomal protein-antibody	2317S; Cell Signaling Technology, Massachusetts, USA	1: 2000
Anti-Phospho- NF-κB p65 (Ser536) antibody	3033P; Cell Signaling Technology, Massachusetts, USA	1: 5000
Anti- NF-κB p65 antibody	8242P; Cell Signaling Technology, Massachusetts, USA	1: 4000
Anti-p53 antibody	2524T; Cell Signaling Technology, Massachusetts, USA	1: 1000
Anti-p21 antibody	10-7526; ABGENEX Pvt. Ltd., Odisha , India	1:1000

Anti-PTEN antibody	11-7539; ABGENEX Pvt. Ltd., Odisha , India	1: 1000
Anti-LC-3B antibody	2775S; Cell Signaling Technology, Massachusetts, USA	1: 1000
Anti-Cox-2 antibody	12282P; Cell Signaling Technology, Massachusetts, USA	1: 2000
Anti-survivin antibody	2808BC; Cell Signaling Technology, Massachusetts, USA	1: 2000
Anti-XIAP antibody	20-1106; ABGENEX Pvt. Ltd., India	1: 1000
Anti-cIAP-1/HIAP-2 antibody	20-1054; ABGENEX Pvt. Ltd., India	1: 1000
Anti-Caspase 9 antibody	9508T; Cell Signaling Technology, Massachusetts, USA	1:2000
Anti-CXCR4 antibody	ab124824; abcam®, Cambridge, USA	1: 2000
Anti-MMP-9 antibody	13667P; Cell Signaling Technology, Massachusetts, USA	1: 1000
Anti-rabbit secondary antibody	ab97080; abcam®, Cambridge, USA	1: 6000
Anti-mouse secondary antibody	ab97040; abcam®, Cambridge, USA	1: 6000
